# Implications of Teacher Life–Work Histories for Conceptualisations of ‘Care’: Narratives from Rural Zimbabwe

**DOI:** 10.1002/casp.2265

**Published:** 2015-12-11

**Authors:** Clare Coultas, Elena Broaddus, Catherine Campbell, Louise Andersen, Alice Mutsikiwa, Claud Madanhire, Connie Nyamukapa, Simon Gregson

**Affiliations:** ^1^ London School of Economics and Political ScienceDepartment of Social PsychologySt Clement's Building, Houghton StreetLondonWC2A 2AEUnited Kingdom; ^2^ Johns Hopkins Bloomberg School of Public HealthDepartment of International Health615 N. Wolfe StreetBaltimoreMarylandUnited States; ^3^ Biomedical Training and Research InstituteNo. 10 Seagrave RoadAvondaleHarareZimbabwe; ^4^ Imperial College School of Public HealthDepartment of Infectious Disease EpidemiologyNorfolk PlaceLondonW2 1PGUnited Kingdom

**Keywords:** care, teacher role, narrative, HIV‐affected learners, Zimbabwe

## Abstract

Schools are increasingly seen as key sites for support to HIV‐affected and other vulnerable children, and teachers are assigned the critical role of identifying and providing psychosocial support. Drawing on the life–work history narratives of 12 teachers in Zimbabwe, this paper explores the psychosocial processes underpinning teachers' conceptualisations of these caring roles. The influence of prolonged adversity, formative relationships, and broader patterns of social and institutional change in teacher identity formation processes speak to the complex and embodied nature of understandings of ‘care’. In such extreme settings teachers prioritise the material and disciplinary aspects of ‘care’ that they see as essential for supporting children to overcome hardship. This focus not only means that emotional support as envisaged in international policy is commonly overlooked, but also exposes a wider ideological clash about childrearing. This tension together with an overall ambivalence surrounding teacher identities puts further strain on teacher–student relationships. We propose the current trainings on providing emotional support are insufficient and that more active focus needs to be directed at support to teachers in relation with their students. © 2015 The Authors. *Journal of Community & Applied Social Psychology* published by John Wiley & Sons Ltd.

## Introduction

International policy and non‐governmental organisations (NGOs) increasingly position schools as sites for pastoral care supporting the wellbeing of HIV‐affected children (Bialobrzeska, Randell, Hellmann, & Winkler, 2009; Sifile, 2010; UNESCO, 2008). However literature supporting this more expansive view of schools is dominated by studies from high‐income countries. Through the life–work history narratives of 12 Zimbabwean teachers we throw a more nuanced light on this literature and the policy it influences by expanding understandings of the psychosocial processes that underlie constructions of caring identities by teachers in vulnerable populations. This has important implications for policy and practice regarding training of teachers as caregivers in HIV/AIDS settings.

UNESCO describes the expanded role for teachers central to this policy vision as supporting children in emotional distress through the provision of counselling, referring neglected or abused children to social services, conducting home visits, making lessons flexible to suit the needs of students who have care duties at home, reducing stigma and discrimination, and generally taking a ‘positive attitude’ to children infected and affected by HIV (2008, pp. 44–49). A growing body of literature however highlights the need for greater attention towards the contextual factors which may undermine the promise of caring relationships in schools in extreme settings (Bajaj, 2009; Hoadley, 2007; Machawira & Pillay, 2009). This article is part of a wider multi‐method study of school support for vulnerable children in Zimbabwe, and two companion papers to this article identify dynamics in six Zimbabwe schools which impede conceptualisations of ‘caring schools’. A story‐creation project with students revealed the importance that children place on the emotional impacts of HIV (as compared to the material impacts which were found to be emphasised by teachers) and the lack of support from school‐based relationships, with students viewing the school as a site of stigma and discrimination more than support (Campbell et al., 2014a). Interviews and focus groups with teachers, parents, and NGO workers in the same sites as part of the wider overall study exposed the realities of already overworked teachers who are themselves affected by extreme poverty and HIV, and the hierarchical poorly resourced institutions in which they are embedded, typified by poor supportive networks within the school as well as with the wider community and external agencies (Campbell et al., 2014b).

Western discussions about teachers' roles increasingly emphasise identity (Goodson, 1992); however, this has yet to be applied to discussions on teachers in extreme contexts. Trevaskis (2006), p. 3 discusses how teachers' perceived identities ‘… underlie their views of the nature of teaching as a profession and the role of the teacher within that profession.’ They indicate the need to move away from essentialist policy conceptualisations of how teachers *should* be and explore the lived experiences and backgrounds of teachers, recognising that the personal and professional are not mutually exclusive nor without historical or contextual basis (Goodson, 1992; Machawira & Pillay, 2009; Trevaskis, 2006; Zembylas, 2003). This is especially important in settings of prolonged adversity and when looking at emotive and moral aspects of teacher–student relationships. Therefore in this paper we aim to explore how teachers in Zimbabwe conceptualise their caring roles and begin to unpack the underlying psychosocial processes that shape this identity formation.

### Conceptual framework

Narratives provide insight into the human and experiential aspects of people functioning in systems and institutions. In healthcare narratives have opened up the ‘lifeworld’ (Husserl, 1970) of the patient as well as impediments to patient–doctor communication (Greenhalgh, Robb, & Scambler, 2006). Lewis stresses that narratives ‘… link personal experience with broader patterns of institutional change,’ and so provide socio‐historical depth through an individual's story (2008, p. 561). Drawing on Ladkin's (1999) conceptualisation of the life–work history [narrative], Lewis builds an argument for the usefulness of narratives in framing the ways people relate with, interpret, and enact social policies in their work (D. Lewis, 2008). In the case of Zimbabwean teachers this therefore involves the unpacking of how changing social and institutional contexts (inclusive of the increasing influence of NGOs) frame how teachers conceive of ‘care’ in their work.

More than this however we propose that the temporality intrinsic to narratives ‘in which past and future cling to the present, and time is experienced [subjectively] as a process of reflection or reflecting,’ (Cunliffe, Luhman, & Boje, 2004, p. 268), opens up exploration of the psychosocial processes which underlie professional (and personal) identity construction. In his life history study exploring teacher motivation, Trevaskis highlights that ‘enabling the individual voice of the teacher to be heard, especially in relation to an issue like motivation, inevitably involves consideration of the person's perceived self identity,’ and the same can be said for the issue of care (2006, p. 2). By invoking memories in reference to the present, narratives speak to the ‘temporal axis of personhood’ (Antze & Lambek, 1996, p. xxv), where the personal, social, and professional interconnect embedded in intersubjective interpretations of the past as well as expectations for the future (Cunliffe et al., 2004). In this way a more nuanced and positioned understanding can be elicited in terms of how ‘historical, political, cultural, societal, institutional, familial and personal circumstance’ (James, 2002) shape teachers' perceptions of their roles as caregivers. We are therefore not making claims as to whether teachers *are* ‘caring’ towards children nor seeking to assert that there is a linear relationship between understandings of care and caring relationships. Rather we are interested in the wider symbolic frameworks in which teacher–pupil relationships are enacted in particular settings.

### Literature review

A large body of literature discusses the importance of ‘care’ in education (Noddings, 1984; Owens & Ennis, 2005; Riconscente, 2014; Rogers & Webb, 1991), particularly for vulnerable and at‐risk youth (Helleve et al., 2011; J. L. Lewis et al., 2012; Mihalas, Morse, Allsopp, & McHatton, 2009; C. Muller, 2001). This predominantly Western literature shapes representations of caring teachers in policy regarding support for HIV‐affected learners. We hope to contribute to the limited empirical literature that studies the application of these policy notions of ‘care’ in lower income countries. In the following sections we review how caring teachers are conceptualised, and then look more specifically at the few studies on caring teachers in settings highly affected by HIV/AIDS.

#### Conceptualising caring teachers (in western settings)

Definitions of ‘caring teachers’ in this literature draw heavily upon Noddings (1984) concept of an ethic of care, which emphasises the importance of understanding the needs of those being cared for and acting in accordance to those needs. Although it has been critiqued from a feminist perspective for perpetuating exploitation of caregivers (Hoagland, 1991), and through a Foucauldian lens for justifying further extraction of uncompensated moral and ethical labour from teachers (McCuaig, 2012), Western teacher education emphasises cultivation of this ethic of care (Goldstein & Freedman, 2003; Rogers & Webb, 1991). Research also demonstrates a connection between caring teachers and positive student outcomes (J. L. Lewis et al., 2012; Mihalas et al., 2009; C. Muller, 2001; Riconscente, 2014).

Noddings asserts that care must be situationally defined rather than viewed as a proscriptive principle (1984, p. 702). Therefore many researchers have examined how caring teachers are conceptualised by students and teachers. Research with teachers has developed the idea of a continuum of care: Franziska Vogt (2002) notes that this continuum ranges from ‘caring as mothering’ to ‘caring as commitment.’ Kemp and Reupert (2012) instead view this continuum as ranging from non‐caring to over‐caring and in their research with pre‐service teachers also describe the importance of individual student and teacher factors as well as school location in understandings of the care continuum. They note the influence of teachers' backgrounds and experiences but provide little exploration of the ways these shape understandings of care. Thompson (1998) draws attention however to the inherent ‘colorblindness’ in mainstream theories of ‘care’ in that they are constructed in terms of the ‘ideal’, based on the privileged position of white parenting where a ‘space of innocence’ (p.527) is created apart from the ‘dangers’ of wider society; a privilege which is not afforded to all. She and others (Seidl, 2007; Tatum, 2007; Thompson, 1998) call for more culturally relevant pedagogies that tap into and foster understandings of differential access to power across racial and class/socio‐economic boundaries.

Multiple studies have noted the important influence of contextual factors like school location and cultural background on understandings of caring (Antrop‐González & De Jesús, 2006; Hayes, Ryan, & Zseller, 1994; Webb, Wilson, Corbett, & Mordecai, 1993). Webb, et al.'s findings suggest that individual biographies and cultural histories play key roles in the process of developing conceptualisations of care. However, their focus is on inter‐group differences rather than examining what these roles or conceptualisations of care are along with how these processes of identity construction develop for individual teachers. Researchers have explored the important influence that teachers' personal histories have on their values and professional decisions (Trevaskis, 2006), and their beliefs about the importance of care (Larson & Silverman, 2005). However, the role of teachers' life–work histories in shaping conceptualisations of care remains unexplored.

Methodologies through which care has been studied range from qualitative and interpretive approaches (Antrop‐González & De Jesús, 2006; Goldstein & Lake, 2000; Isenbarger & Zembylas, 2006) to questionnaires that take care as a predefined concept, a characteristic attributable to individual teachers in varying quantities (J. L. Lewis et al., 2012; Riconscente, 2014; Teven, 2001). Notions of care that appear in policy lean towards these more individualist and static constructs. For instance the UNESCO guidance document on HIV/AIDS and supportive learning environments stipulates that if teachers are trained (through manuals) they will be able to recognize and support students to deal with ‘grief and loss, anxiety and fear about the future, isolation, stigma, and discrimination.’ Barriers identified are a lack of training, lack of time, ‘lack of guidelines about roles and responsibilities’, and a lack of space (2008:47–8). UNESCO's conceptualising of care in this essentialist, proscriptive, and disembodied framing serves the purpose of endorsing solutions such as child‐friendly schools that can be implemented across diverse contexts based on normalising behaviour change training activities. However, a more nuanced understanding is needed about the potential situational and interactional barriers to this policy vision of care being operationalised.

#### ‘Caring teachers’ in extreme settings

Much of the literature on caring teachers in extreme settings, such as contexts of high HIV prevalence, centres around the material/structural challenges faced by teachers in attempting to take on a caretaker role as proscribed by policy (Hoadley, 2007; Machawira & Pillay, 2009; Theron, 2009) and documents the critical need for better training and resources for teachers if they are to play that role (Hattingh & De Kock, 2008; Henning & Chi, 2012; Holderness, 2012; Sefhedi, Montsi, & Mpofu, 2008; van Wyk & Lemmer, 2007; Wood & Goba, 2011). Only a few studies have examined how teachers themselves conceptualise their role as caretakers, and how care and support are operationalised in these settings.

Hoadley and Ensor (2009) demonstrated how South African teachers' own backgrounds shape their conceptions of their roles as teachers, but no studies have yet explored this topic in the context of Zimbabwe, or looked specifically at teachers' conceptions of their roles as caretakers for HIV‐affected children. van Wyk and Lemmer (2007) interviewed teachers in South Africa regarding the needs of their students and types of support that they provided. Many reported little knowledge of their students' home‐lives, saying that for students to share this kind of personal information with teachers was not customary, that many families wished to keep their struggles a secret, and moreover that many students recognized that teachers themselves were struggling financially and therefore would be of little assistance. They noted, however, that many teachers did mention providing students with material support like money for school fees, school uniforms, and food. Both Bajaj (2009) and Bhana, Morrell, Epstein, and Moletsane (2006) examined contextual (such as economic and administrative) factors that either facilitated or inhibited caring relationships in Zambia and South Africa respectively. Concurring with the findings of the companion papers to this study (Campbell et al., 2014a; Campbell et al., 2015; Campbell et al., 2012), many of the examples of support described by teachers were monetary or material, clashing with students who emphasised more the need for emotional support (Campbell et al., 2014a).

Only one previous study (Machawira & Pillay, 2009) of caring teachers in extreme settings takes a narrative approach to explore how teachers understand their roles as teachers and caretakers. They discuss the narratives of HIV positive teachers in Zimbabwe regarding their illness, and demonstrate how these teachers' own struggles with HIV make it difficult for them to play the caring role for HIV‐affected children that is envisaged by policy. This study illustrates the value of taking a narrative approach, and we work to build upon the insights provided through a wider focus. Specifically, we explore how life–work histories in prolonged adversity, which in Zimbabwe in addition to HIV/AIDS includes violent conflict and more recent economic and political tumult, frame teacher–student ‘caring’ relationships. We posit that this will provide a subjective, interactional and contextualised perspective on the challenges to implementing policy conceptualisations of care in extreme settings, and will contribute more nuance and non‐Western perspectives to the wider literature on theories of ‘care’ in schools.

## Method

This paper draws on life–work history narrative interviews with a total of 12 teachers—one female and one male per school from each of three Primary and three Secondary schools at three different sites (one Primary and one Secondary school per site). Interviews were conducted between July 2012 and May 2013 as part of a larger multi‐method study looking at the role of schools in supporting the inclusion and well‐being of children affected by HIV/AIDS in rural Zimbabwe. For the larger study, 18 teachers were recruited for in‐depth interviews by approaching the headmasters at each of the six schools, requesting their approval for the school's involvement in the study, and then asking them to identify three teachers to interview (including at least one male and one female). After the in‐depth interviews, two (one female and one male) of the three teachers from each school were then invited to participate in narrative interviews, which occurred several months later. When deciding which of the teachers to invite, study staff selected those that had been more open and elaborating during the previous interview. The 50–50 male to female ratio is roughly typical of the teaching profession in Zimbabwe, where Primary school teachers were 55% female and 45% male and Secondary school teachers were 45% female and 55% male as of 2012 (The World Bank, 2015). Unfortunately, information on contextual factors, such as years of teaching, was not collected from other (non‐interviewed) teachers at each school, so it is difficult to know how ‘typical’ our group of interviewees is. It is possible that some headmasters identified more experienced or better performing teachers for interviews, as compared to other teachers at the school.

Study staff explained to teachers invited for narrative interviews that the interview would focus on their own personal life story, as the study sought to fully understand how their backgrounds influenced their current position as teachers. All interviews were conducted by Zimbabwean research assistants in the native language of the interviewee and lasted an average of 65 min. Research assistants then transcribed and translated each narrative interview. All research assistants held university‐level social work degrees and had received training in qualitative research methods and research ethics, as well as in‐house training from more experienced researchers. This included training on the informed consent process (written consent was gathered from each participant) as well as protocol specific to the narrative interviews. Interviewers encouraged participants to speak as freely as possible without interrupting, listened respectfully throughout, and did not probe on sensitive topics but rather let the types of experiences and level of detail shared be at the discretion of the interviewees. The project received ethical approval from the Medical Research Council of Zimbabwe (MRCZ/A/1661) and the Research Ethics Committee at The London School of Economics. Interviews focused on experiences of work and the surrounding ‘social networks and settings’ (D. Lewis, 2008). For instance participants were asked to talk about their parents' education, family relationship, and ties to community; the good and bad experiences throughout their education; their reasons for wanting to become a teacher and what they love and find difficult in their career; as well as their perceptions of relationships in the communities where they work and live. Data were analysed by the three lead authors using Muller's (1999) pragmatic approach which applies an inductive thematic analysis, identifying relevant patterns to the research question across the transcripts while ensuring that data are not taken out of the context of each individual narrative. In this way each teacher's narrative was analysed as whole but also in connection with the narratives of the other teachers according to a two‐pronged approach.

First, a detailed thematic analysis was conducted eliciting 30 themes relevant to the research focus. Examples include *important adult figures during childhood*, *learning values*, *being disciplined*, *advantages*/*challenges youth today face*, *changes in the value of education*, *desire to become a teacher*, and *lack of respect as a teacher*. Through discussion these were then grouped into six overarching themes (*role models*, *conflicting representations of childhood*, *character classifications*, *material deprivation vs*. *access*, *devaluing of education and teacher identities*, and *poor social capital*) that were used to frame a return to the analysis of each individual narrative as a whole exploring its temporality, particular narrative constructions used, as well as the identification of similarities and differences across narratives. Further discussion around this analysis then generated the three segments laid out in the results, and findings were shared with all other authors inclusive of field researcher assistants for contribution. All teachers interviewed have been given pseudonyms to protect their anonymity, and where necessary reference to their gender has been omitted.

Although we were unable to conduct member‐checks of our findings, we sought to ensure the authenticity of our data and conclusions throughout the research process by attending to the fairness of the data collection and analysis process and the evocativeness of the findings presented (Guba & Lincoln, 1994): Particularly within the scope of the larger multi‐method research process (in which students, parents, administrators, and other community members were interviewed as well as teachers), we sought to access and balance a wide range of participant perspectives, and during the narrative interviews research assistants encouraged teachers to speak freely, clearly communicating that their opinions and experiences were valued. As described above in the analysis section, themes identified were grounded in the narratives, and we looked across all narratives to understand the range of perspectives on a given theme, seeking to fairly represent the diversity and complexity of our participants' experiences. Finally, the Zimbabwean field workers included in our team of co‐authors helped to ensure that our findings and presentation of them were evocative of local realities and understandings.

## Results and Discussion

Teachers' life–work history narratives uncovered striking differences in conceptualisations of care in teaching, not only with the conceptualisations that appear in policy and Western literature on ‘care,’ but in comparison to one another. There was no clear pattern to these individual differences for instance by gender, school level of teaching, or location (e.g. urban/rural) although this could be a limitation of the small sample size. Yet patterns could be seen in the psychosocial processes found to underpin conceptualisations of care related to interpersonal relationships and the juxtapositioning of one's own childhood with the present day. We also noted patterns in teachers' strategies for reconciling identity, disrupted by broader patterns of social and institutional change. We discuss each of these in turn.

No questions were asked regarding the teachers' HIV status; nevertheless, two of the 12 teachers disclosed that they were HIV positive. While one appeared to be functioning quite well in the workplace, the other expressed feelings of distrust with their colleagues and an unhealthy work environment. Considering that one third of teachers in Zimbabwe are thought to be infected with HIV (Shizha & Kariwo, 2011) it is possible that more of the participants were HIV positive however did not disclose.

### Conceptualising ‘care’ in teaching

The narratives indicated a strong yet complex connection between formative childhood experiences and relationships of support (or lack thereof) and the ways teachers position themselves as caregivers to their students. All recalled adult role models whose behaviour, actions, or instruction played an influential role in their life. The types of care that they described receiving from such role models, seeing them provide to others, or in some cases wishing that they had received, were mirrored in their descriptions about how they viewed their caring roles as teachers. We have broadly grouped these into three different conceptualisations of care which we will unpack in turn: care as material support (which dominates the current body of literature on caring teachers and HIV), care as discipline, and care as love and emotional support (prioritised in policy). Unlike Vogt (2002) and Kemp and Reupert (2012) we do not identify these various forms as being organised across a continuum. Rather our examination of the teachers' narratives indicates that these fundamentally different dimensions to care interact and overlap in a range of different ways, with particular blending around emotional care. It is perhaps of no great surprise that in a context of widespread poverty, material support was the most prevalent understanding of care. Eight teachers emphasised their care for students through the provision of money for school fees, shoes, school materials, and food; and all described support from their own caregivers in this same way. Five teachers talked about the importance of discipline, emphasising their desire to help children be successful in life by instilling in them a commitment to hard work, seriousness about their studies, and respect for elders.

‘When we were growing up it was a very good time. The way today's parent is bringing up his child and the way our parents and teachers brought us up, we see that things were good in the past. Discipline should be found in every child, a good citizen. Nowadays [they say] that a child should not be touched, but we were beaten.’—Emmanuel, Primary Level Teacher, 49 years old

Six teachers (two male; four female) described a conceptualisation of care as ‘loving children’ and providing emotional support. However half of these teachers emphasised only material or disciplinary aspects when giving examples. In the following excerpt, Daya, a 48‐year‐old teacher at the secondary level, explicitly notes that loving and caring does not necessarily require material support, yet elaborates on these statements with only examples of material support:

‘I think that a good teacher is someone who empathizes with a sick child involuntarily, it just happens. Helping does not mean that you have to give materially. Helping is love. You can help a sick child by taking him to the clinic…. You care about him. If he does not have a book, you see if there is anything that you can give him.’

The narratives of the three participants who discussed care as it is envisaged in policy and Western theories of ‘care’, such as loving children, taking on a parental role, and counselling students about issues in their home‐life, demonstrate clearly the complexity and nonlinearity of the processes connecting formative experiences of care to current conceptualisations. In fact, each of their narratives shows the ways in which different coping mechanisms or a contradictory intermingling with other conceptualisations of ‘care’ can limit the potential for teachers to provide love and emotional support. We discuss each of these in turn.

*The Functioning Caring Teacher*: Peter, a 34‐year‐old primary school teacher, describes a challenging childhood, yet to a far greater extent than the other teachers he frames his recollections with an emphasis on the emotional aspects of care and support, or the lack thereof. His parents divorced when he was young and he was raised by his grandmother who struggled to provide food, clothing, and money for school fees. He recalled wishing that he had received more emotional support from his parents as a child and talked extensively about the support his grandmother provided him: ‘Everything she got she shared… she tried to give me enough love as my grandmother. I think she played an important role in my life… She motivated and comforted me.’

This encompassing of material support in more emotional aspects came out when he talks of his own students. He mentioned providing material support like books and pens, but emphasised the necessity of an emotional and trusting relationship in order to know his students' specific needs.

‘By loving the children that you teach, the children will learn to trust you. When children trust you they open up about certain things that you never thought of. Here we had cases where we heard about children that are sexually abused… Those children start learning that I can trust my teacher and open up as they talk to the teacher. So I think a teacher that is good at his job must be close to his or her pupils. Know them, love them. Love does not have limits.’

Throughout his interview Peter empathised with orphans and the grandparents who struggled to provide for them:

‘Most of the guardians we see here are grandparents who come to ask that the school allow their grandchild to be let in until they can work out something to raise the fees. That is very important to me. That love that is in grandparents for their grandchildren although they themselves do not have much and need support. That is important to me, maybe because it is similar to my background.’

And he explained the ongoing effects of emotional problems from his background:

‘Regarding emotional health, yes, I think there was a time I was found wanting. Growing up there are things that one wishes for in life. Those may be emotional depending on your thinking, and this may affect your health… . There are certain opportunities that I missed growing up that I should have gotten. Sometimes it haunts us. When you try to look at where we are in our lives. Then you start to draw back and think had you had both parents with you maybe you would have turned out differently… But we sometimes manage to deal with the stress and the emotions and learn to go on.’

Peter explicitly associated surviving childhood difficulties with the care and emotional support that he got from teachers and related his desire to become a teacher to wanting to do the same for others.

‘I only understand it now that as a teacher you can be influential. Through that experience that I had in life… They were people who accepted us and knew our backgrounds and where we came from. They tried to accommodate us when they were teaching… if it had not been for education or for my teachers I would not be at the position that I am now. So I have a desire to do the same for others.’

*The Tortured Caring Teacher*: The narrative of Jendayi, a 39‐year‐old primary school teacher, demonstrates connections between early experiences of care, and current conceptualisations, but also how teachers' own histories of trauma may impede their ability to take on emotionally supportive roles for students. Jendayi suffered severe physical and sexual abuse growing up in the household of her half‐sister. Although her parents neglected her both materially and emotionally, she described receiving emotional support from an aunt who, ‘encouraged me to work hard and told me that one day I will leave this suffering,’ and her teachers who, ‘loved me because I was intelligent and also because of my home situation. The teachers loved me so that other children could accept me.’

She emphasised the importance of loving her students and of teachers making themselves available by being friendly and open. Yet, her comments also indicate the burden of struggling to care for children dealing with issues like the sexual abuse that she encountered in her own troubled past, and highlights that in extreme settings many teachers are themselves in need of care:

‘Sometimes we have counselling lessons alerting children about abuse and rape issues … [but] sometimes you might fail to counsel the child because you will also be in the same situation. You will also be in need of a counsellor… Let me give the rape cases as an example. You might also have been involved [in a similar situation] and have it affecting you. So for you to be able to counsel a child… When you are also in the same situation… it is really complicated.’

*The Contradictory Caring Teacher*. The third teacher who emphasised emotional support is Faith, a 37‐year‐old primary school teacher. She too explicitly connected her conceptualisations of care to her upbringing, specifically her mother:

‘I am that kind of person who feels for others especially if they are facing challenges. Like in my class I have children who should not be here [because they have not paid school fees] but who I told not to go anywhere when other children are being chased away… So I was thinking that if I did two or three projects maybe I will be able to pay half of the fees for one of these kids without the grandmother knowing… Because that is what I was taught and it's not like I have to think about helping. I inherited that from my mother…And I wish I could counsel them so that they can be happy. I may not have anything to give them. But maybe just a few words that I can give them or tell them of my experience if they have similar problems to mine. Maybe they will see that there is still hope.’

Faith's comments, however, indicate the pervasiveness of operationalising care as discipline, which can actively work against care as love and emotional support. She emphasised the importance of discipline through corporal punishment for helping children live up to their potential:

‘Once these children know that they will not be beaten, even a child who has the ability, who is capable, may not complete their work every day saying that they don't have a pen or hiding their book… Because they know there is nothing you can do to them, they will not try to improve. It's different from a child who knows that if I do this Madame will not like it. The child will try by all means to please you so that they will not be beaten. In pleasing you they are lifting themselves up.’

She even admits to beating a girl (despite it being illegal in Zimbabwe) ‘who started being naughty [having sex] at an early age’, and views her action in this event as equating to surrogate parenting—‘… that is why I have beaten her. To show her not to boast with immoral behaviour and that I am the parent.’

### Ideological dilemmas and nostalgia in constructing childhood

Faith's positioning of herself as the parent and so being justified in beating her student is rather different from policy visions of teachers loving children like a parent, and speaks to a much wider tension surrounding the guidance and control of children. Frankenberg, Holmqvist, and Rubenson (2014) describe this ‘ideological dilemma’ (citing Billig 1988) as arising from conflicts between the (Western) individualist ideology of child rights and participation versus more community‐situated practices built on protection through authority. They highlight how the latter childrearing ideology has been broadly disrupted by communities becoming more diverse and accordingly detached as well as by global influences through technologies (ibid). Their work did not extend to schools yet these same tensions can be seen in the narratives, with all teachers drawing sharp contrasts between their own childhoods and the conditions and upbringing of today's children, centred on the matter of discipline.

The majority of teachers viewed their students as having an easier life and as being less disciplined, however even those who acknowledged the difficulties faced by youth today emphasised the importance of learning key character traits as a child, such as discipline, respect, and love of learning. These were traits that all of the teachers credited with their success in school and in life. The majority, eight, framed descriptions of their students using these same character traits, describing them as disciplined, focused, and studious, and thus deserving of support, or instead as playful, mischievous and lazy and therefore not deserving. Their comments indicate a prevailing view of learners' success or difficulty in school as directly related to character and initiative. For example, Gamba, a 39‐year‐old male secondary teacher, who viewed ‘care’ as discipline, explained:

‘You asked about the quality of education in our schools? I'm still comfortable with it. I blame our students, but our education is at a high standard. I usually blame the students, because they are not serious.’

These teachers frame their role as teaching students positive character traits in order to help them be successful in the future, as they learned from their role models. This clear prioritisation by all teachers of needing to prepare students through discipline for the difficult life ahead of them speaks to Thompson's (1998) argument about pedagogies not rooted in white privilege. Yet a focus on these individual character traits can make teachers dismissive of certain children as undeserving or as not wanting education, and shifts more of the responsibility to students for their education, away from contextual factors and also the potential role that teachers can play in assisting students to deal with difficulties outside of their control. When asked about the quality of schools she attended while growing up, a 43‐year‐old secondary teacher named Angeline explained that the teachers were good, and therefore, ‘those who wanted to learn would learn.’ In line with her view of ‘care as material support’, she criticised the way that NGOs select students to support with funding for school fees, explaining that the money often goes to children that ‘do not care about school.’

Only three of the 12 teachers interviewed (none of whom experienced childhood trauma or contextual violence such as war or riots) discussed external factors in addition to individual character traits as determinants of children's school performance. These teachers discussed the significant challenges faced by today's children because of poverty and the destructive effects of HIV/AIDS, which impact their behaviours and abilities. However, two of these three, in accordance with their conceptualisation of ‘care,’ spoke primarily of material deprivation. The exception to this again was Peter, whose conceptualisation of care as emotional support is reflected in his description of the external challenges faced by students:

‘Teaching from my point of view is not merely standing in front of the class, writing on the chalkboard and telling children to write the work. You have to focus on quite a number of things. You have to motivate the children that you teach, know their background and why is she performing the way she is. Somethings might be related to the family she comes from. You find that there is a lot of information you need in order to deliver a lesson because you are delivering a lesson to different pupils with different backgrounds. Therefore even though there is natural IQ where some children were born with natural intelligence, there are factors which affect IQ. You find that for a child coming from a disadvantaged home, sometimes it takes time for them to start concentrating on what is being said. Then when you ask you find out that they had not eaten. And they are thinking of what to do, and of the tasks that await them when they get home.’

In contrast, the teachers (over half) who recalled living through childhood traumas such as outbreaks of war or riots were the most vocal about how today's children are lazy and unappreciative. For example, the narrative of Canaan, a 46‐year‐old secondary school teacher, demonstrates how this group of teachers, despite associating their own childhood with violence, are nostalgic for a past that is romantically ‘reified in relation to the disillusioned present’ (Duncan, Stevens, & Sonn, 2012, p. 209):

‘When we were growing up, my childhood was terrible because it was a time of war. Learning was also a challenge because we spent time without going to school. I spent two or three years disconnected and not learning because of the war… When we were coming from school, we sometimes encountered the comrades… So we were rather traumatized. I can say it disturbed us, like when there were gun shots, bombs, and so on. Sometimes the sounds would be so close… But on morals, life was good… Mischievous children? There were no great levels.’

This ‘disillusioned present’ was largely seen to be rooted in Western influences. Canaan (who interestingly conceptualised ‘care’ as material support) for instance talked about how ‘behaviours have depreciated’ with increased access to technologies. And half of the teachers talked about how child rights policies and the banning of corporal punishment are impeding their ability to discipline and adequately care for their students. One of them, Angeline, critiqued the impact of the child's rights movement (perceived as a Western idea), framing it as, in the end, doing a disservice to today's youth:

‘When we went to school, things were very difficult. Back then for a child to be disciplined a stick would be used. Right now they say you talk to a child using other ways apart from beating. Also, children have a better life. Nowadays it is better. If you look at the conditions that I grew up in as compared to these children, it is different… The way of living back then was difficult. We worked extra hard. The children's rights are preventing certain things from being done to children. But on the other hand, some of these things are causing them to fail to focus on what's ahead.’

### Teacher identities in ambivalence and broader patterns of change

Duncan et al. (2012) identify how nostalgia is a personal and collective process that represents a loss, disappointment, or ambivalence in the present. Certainly a strong ambivalence can be seen in the narratives, not only between different forms of childrearing but also surrounding teacher identities. All participants talked extensively about the 2008 inflation crisis that dramatically decreased the salaries of Zimbabwe's teachers, doctors, and other professionals, with nine out of the 12 explaining that it caused a sudden decline in the social status of teachers and a devaluing of education more generally.

As Gamba explained:

‘I started teaching in 1997. Students were well behaved and they passed… But when the issue of inflation began, they no longer saw the value. They started becoming negligent and they would say, ‘Why should I learn? Education is not well paying. We will look for money when we hustle.’ That spirit affected children very much up to today. It reduced the value of education and it spoiled these children. They were all still in primary during the inflation period in 2008 but they heard about it and heard their elders saying, ‘Why would you become a teacher or a doctor?’ Even the doctors were mocked… So respect for educated people was reduced. And when something's value has been reduced, it is difficult to increase it.’

The three teachers who did not describe changing social perceptions of teachers, despite describing dissatisfaction with the amount of pay received, in the face of this dilemma, reasserted their love of their job, their belief in the importance of education, and their belief in the necessity of what they do for children. Peter, who described becoming a teacher because it was his calling to help children the way that his teachers had helped him, discussed:

‘Sure if there's a job that can be despised because of its salary, it's teaching. But there is no greater joy than seeing your student working in a bank, seeing your student wanting to thank you for what you taught them…. It actually surpasses getting a huge salary, just knowing that I had a hand in a certain child's life.’

Angeline, who explained that she became a teacher in order to help children understand the value of education and to improve their lives, commented,

‘It takes time for the school fees to be raised and there is too much work so at times you will be demotivated. But because you have children at heart, you will work in order to help the child.’

However for all other nine teachers, the broader patterns of social and institutional change have quite clearly contributed to what Duncan et al. (2012) (drawing on Freud) describe as ‘melancholia’. They describe this form of nostalgia as relating to a loss of location or lack of coherence in identity (ibid), and this can certainly be seen in the narratives of teachers not driven solely by ‘helping children’. Canaan for instance described getting into teaching because ‘… teachers were highly regarded. All professionals of the day, even in town, people would give salutations to a teacher… We were driven by money. Those days teaching had money… [Now] I hope to relinquish teaching and relocate. Yes, may God help me to leave teaching and enter into another field that has variety.’

In fact, of the seven teachers who described wanting to leave the teaching profession, only one gave solely financial reasons, with all others, like Canaan, framing this desire in terms of feeling that they haven't achieved their full potential. Interestingly, for many accomplishing more meant obtaining further education themselves, as Jacoline, a 38‐year‐old secondary teacher, explained:

‘My hope is for me to be able to do something. My heart tells me that I do not want to die as a teacher. But I worry that I will not be able to study so that I can get better qualifications that will pull me out of the education system.’

Duncan et al. (2012) stress that the ambivalence of the melancholic position opens up possibilities for new and more complex identity formations. And certainly this strong desire expressed by all teachers of wanting to further themselves is a potential resource and strategy for this. However, this is quite clearly not being tapped into as many described feeling devalued by those in charge at their workplaces and by the various NGOs working in the schools. We propose that this neglect of teacher aspirations can be seen to exacerbate ‘professional melancholia’ and contribute to resentment towards students, immediately foreclosing potential for empathy and emotional support. Canaan for instance described a lack of appreciation on the part of students: ‘We contribute a lot, we teach these children, they don't plough back. We teach them, some of them we buy things for them. Then they pass and go forward, and when they see you they don't have the attitude to give back to the school.’ Whereas Emmanuel talked about how child rights by giving power to children, takes power away from the teacher, saying ‘the administration continues bringing new policies and these days they love the child so much more than the teacher. This child is now ahead of the teacher.’

## Conclusions

These life–work history narratives open up insights new to the existing literature on ‘caring teachers’ in extreme contexts. Quite clearly conceptualisations of care are embodied and situated, connected in complex ways to formative childhood experiences and strategies for reconciling the past with the present and future in both personal and professional identity processes. In extreme contexts where teachers have themselves lived through and continue to struggle with prolonged adversity and insecurity, a view of ‘care’ can be seen in which the primary focus is on survival and the discipline needed to do this, more than nurture and emotional support. And the teachers quite clearly identify themselves in this ongoing struggle for survival and betterment along with their students. In this way, not only do we see the majority of teachers not buying‐in to policy visions of care, the narratives indicate that child rights discourse (together with broader patterns of institutional and social change) can instead exacerbate feelings of neglect amongst teachers and their resentment towards and blaming of students. As Cockburn (2005) unpacks, the discourse of ‘rights’ in regards to children often denies the ‘contested nature of care’ built upon asymmetrical relationships, and the according contradictions that ensue. Clearly the barriers to [emotional] caring teacher–student relationships in contexts such as Zimbabwe run much deeper than UNESCO's identification of a lack of training, time, space, and guidelines (2008). As Frankenberg, Holmqvist and Rubenson (2014) highlight ‘It is in relation to local ideology that the discourse of child rights needs to be understood by professionals as well as caregivers themselves, as it is this local ideology that provides the foundation for caregiving practices based on child rights’ (p.202–3). In order to create space for emotional care in school settings, the material and disciplinary aspects to care need to be acknowledged, unpacked, and integrated in some way, and situated in the wider ideological dilemma surrounding childrearing. As suggested by Cockburn (2005), shifting discussions from an ‘ethic of rights’ based on universal rules and claims, to an ‘ethic of care’ focussed on contextually specific relationships and responsibilities could be a starting point for this.

More than just differences in conceptualisations of ‘care’, however, the life–work history narratives exposed how the intermingling of personal and professional identity processes can limit the capability of teachers in providing emotional support to their students. Despite the small sample size it is important to note that all teachers who talked about past trauma and violence contrasted today's children in a negative framing and also failed to recognise the impact of contextual factors such as HIV/AIDS and poverty on their students' lives and their ability to do well in school. This nostalgia for the past and view of the present day as being easier (and ‘failing’ children as ‘lazy’) not only reduces the potential for empathy that Noddings (1984) identifies as being essential for a caring relationship, but also could impede a teacher's ability to identify vulnerable children for referral to other resources and services (as envisaged in policy). Certainly the pervasiveness of exposure to violence as seen in the narratives speaks to the importance of ensuring reflective space for the past or present suffering of teachers in any ‘care’ programmes implemented in extreme settings. And recognising that there are different forms of adversity and according coping strategies is also crucial to this.

The life–work history narrative method has therefore not only exposed different conceptualisations of ‘care’ but through temporality – the reconciling of past, present, and future – also provides insight into the psychosocial processes by which interpersonal relationships and broader patterns of social change underpin the ways in which teachers view their caring roles. We were limited by the logistics of the larger study in which these narrative interviews were situated in not being able to member check our findings, and identify a number of ways in which the method could be expanded on for future study. A more longitudinal focus for instance could elicit how understandings of care might change over time and perhaps even in reference to interventions at the institutional level. The more synchronic aspects to narratives, i.e. how the researcher and participants co‐construct knowledge specific to the interview, could also be unpacked further with a series of interviews. Certainly our findings support the view that teacher identities, which by nature incorporate personal experiences and constructions of childhood with professional motivations, are key to any discussion about ‘care’. The dynamic nature of identities and transformative possibilities opened up by for instance unpacking nostalgia could work as useful starting points for programmes aimed at building emotional support in schools. Not only would this facilitate ‘care’ for teachers as well as students, greater understanding could also be gained on the specific ‘cultural pedagogical’ needs of schools in extreme settings.
